# Arrhythmogenic Cardiomyopathy: Expanding Phenotypic Variability in Different Populations Around the World

**DOI:** 10.1016/j.jacasi.2025.05.010

**Published:** 2025-07-01

**Authors:** Natalia Olivetti, Luciana Sacilotto, Marcelo Luiz Campos Vieira

**Affiliations:** a Laboratory of Genetics and Molecular Cardiology, Instituto do Coração (InCor), Hospital das Clínicas HCFMUSP, Faculdade de Medicina, Universidade de São Paulo, Sao Paulo, Brazil; b Precision Medicine Departure, Hospital Israelita Albert Einstein, Sao Paulo, Brazil; c Arrhythmia Department, Instituto do Coração (InCor), Hospital das Clínicas HCFMUSP, Faculdade de Medicina, Universidade de São Paulo, Sao Paulo, Brazil; d Echocardiogram Imaging Department, Instituto do Coração (InCor), Hospital das Clínicas HCFMUSP, Faculdade de Medicina, Universidade de São Paulo, Sao Paulo, Brazil; e Echocardiogram Imaging Department, Hospital Israelita Albert Einstein, Sao Paulo, Brazil

**Keywords:** arrhythmogenic cardiomyopathy, cardiomyopathy, epidemiology

**Figure:**
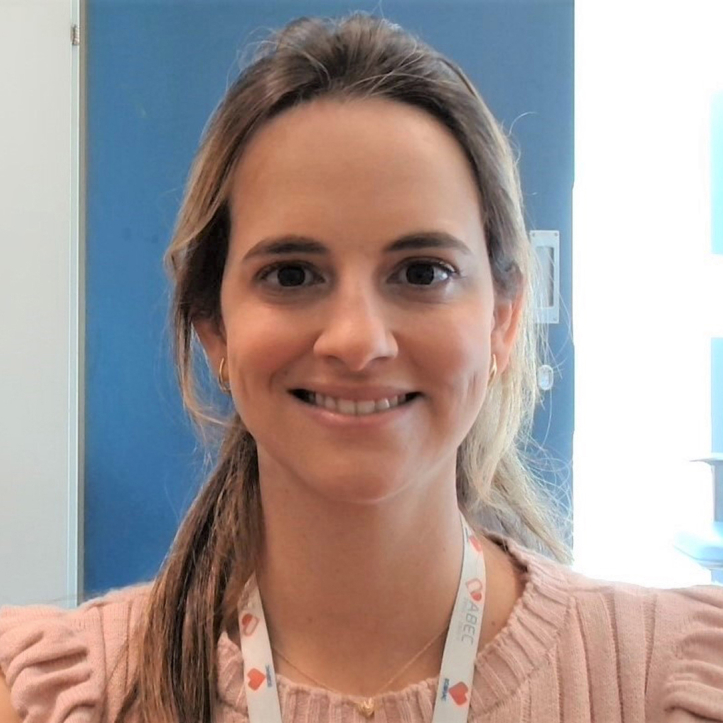

Arrhythmogenic cardiomyopathy (ACM) is a hereditary cardiomyopathy caused by genetic variants in genes encoding for desmosomal or nondesmosomal proteins.^1^ Desmosomes are specialized structures critical for cell-to-cell adhesion between cardiomyocytes.^1^ Disruption in desmosomal assembly facilitates the nuclear translocation of plakoglobin, which shifts gene transcription from myogenesis toward adipofibrogenesis.^2^ This process ultimately leads to fibrofatty myocardial replacement,^2^ causing a vulnerable substrate for ventricular arrhythmias via anatomic re-entry.^1^ Considering clinical aspects, ACM manifests as ventricular arrhythmias, arrhythmic syncope, and even sudden cardiac death (SCD).^1^ It predominantly affects young individuals, with an estimated prevalence ranging from 1 in 2,000 to 1 in 5,000.^3^ Notably, ACM is one of the most important causes of SCD in young people, especially athletes.^4^^,^^5^

The only proven life-saving intervention in ACM is implantable cardioverter-defibrillator therapy.^1^ Antiarrhythmic medications and catheter ablation are adjunctively therapies used to reduce arrhythmic burden and improve quality of life.^1^ However, as disease progresses, patients may develop ventricular dysfunction and ultimately heart failure, a growing concern in the long-term management of ACM.

Although initially described as affecting only the right ventricle, ACM has long been recognized to often involve the left ventricle (LV), and in some cases, involve predominantly the left-sided disease.^6^ This evolving understanding led to the renaming of arrhythmogenic right ventricular cardiomyopathy (ARVC) to ACM, and to major revisions of diagnostic criteria. The 2010 International Task Force criteria primarily addressed the right ventricular (RV) phenotype.^7^ The subsequent Padua criteria expanded the diagnostic framework to include LV-predominant forms, emphasizing myocardial scar patterns detected by cardiac magnetic resonance, especially the characteristic ring-like subepicardial or midmyocardial fibrosis visualized by late gadolinium enhancement.^8^^,^^9^ Most recently, an alternative proposal by the 2024 European Task Force consensus integrated criteria for diagnosing right, left, and biventricular forms of ACM.^9^

Genetic testing plays a pivotal role in confirming the diagnosis and informing clinical management. Genotype-phenotype association has been described in ACM: variants in desmosomal genes—*PKP2, JUP, DSG2, DSC2,* and *DSP—*are most commonly implicated in RV-predominant disease. In contrast, LV-predominant forms are more frequently associated with *DSP* and nondesmosomal genes such as Fosfolamban (*PLN*), Desmin *(DES*), Lamin *(LMNA)*, Titin (*TTN*), and Transmembran Protein 43 (*TMEM43*).^10^, ^11^, ^12^ Emerging genotype-phenotype associations further reinforce the need for individualized evaluation strategies.

Over the past decade, substantial efforts have been made to improve risk stratification for arrhythmic events.^13^^,^^14^ The seminal study by Mazzanti et al^14^ systematically evaluated clinical predictors of life-threatening arrhythmic events in ARVC patients, identifying variables such as male sex, syncope, atrial fibrillation, sustained monomorphic VT, and postdiagnosis intense exercise as independent risk factors. Subsequently, clinical prediction models, such as those developed by Cadrin-Tourigny et al,^15^^,^^16^ have identified age, sex, extent of T-wave inversions, and ventricular function as key predictors. Together, these studies laid the foundation for further efforts toward personalized approaches based initially on clinical markers.

However, these models were primarily based on Western cohorts and mostly population from Europe and North America; thus, their generalizability to non-Western populations remains uncertain. Genetic heterogeneity across worldwide populations may interfere with the prevalence of pathogenic variants, epigenetic regulation, clinical expression, and environmental interaction, ultimately influencing clinical risk predictor of each cohort.

Nowadays, the arrhythmic predictors are better understood. Once arrhythmic events are prevented and have controlled arrhythmic burden, in a considerable number of patients the disease progresses to ventricular dysfunction, leading to ventricular dysfunction, heart failure symptoms, and even end-stage heart failure and death. Progressive heart failure is an important contributor to mortality in ACM patients. Despite this, data on predictors of heart failure–related outcomes in ACM remain scarce.

In this context, the ChinaCORE ACM Registry (China Multi-center Cohort Study on Risk Evaluation of Arrhythmogenic Cardiomyopathy), published in this issue of *JACC: Asia*, represents a major milestone. Hu et al^17^ described a large, national ACM patient cohort that aims to contribute for future risk prediction research. This national registry includes 622 patients, 552 probands with definite or possible diagnoses, and 70 relatives. Notably, the cohort exhibited a median age of 33 years at diagnosis, a high frequency of T-wave inversions in leads V_1_ to V_3_, and a relatively high prevalence of epsilon waves (24.8%).^17^ Moreover, patients presented with a relatively lower LV ejection fraction at baseline.^17^ During follow-up, 40.1% experienced arrhythmic events and 21.9% developed heart failure.^17^ These characteristics demonstrate specific features of distinct phenotypic characteristics of the Chinese population, likely driven by a unique genetic background. The ChinaCORE ACM Registry is not only one of the largest ACM cohorts to date from Asia, but also provides critical insights into risk stratification, genotype-phenotype correlation, and clinical outcomes. Importantly, this study underscores the urgent need to examine ACM within diverse populations to enhance global understanding of its natural history and regional variability. Furthermore, it reminds us of the broader importance of understanding geographic and ethnic variability in cardiomyopathies, an essential step in global cardiology.

In Latin America, for instance, the Brazilian ACM cohort reported by Olivetti et al^18^ included 111 patients with definite diagnosis of ACM. The mean age at diagnosis was 36.3 years.^18^ Most patients were men (72%) and probands (83.8%), more than one-half of patients had sustained VT at the time of diagnosis (56.8%), and 7.2% had aborted SCD at diagnosis.^18^ HF symptoms were present in 18.9%.^18^ During follow-up, 30% experienced arrhythmic events and 10% developed end-stage heart failure outcomes.^18^ Their data revealed that low QRS voltage was a strong predictor of heart failure death and transplant need, with each additional lead showing low voltage, increasing the risk of life-threatening arrhythmias by 17%.^18^ Another study from the same group demonstrated that an RV free wall longitudinal strain value below 14.35% (absolute) was significantly associated with arrhythmic outcomes.^19^

Both the Chinese and Brazilian cohorts reported a high prevalence of heart failure, including a substantial proportion of patients progressing to end-stage HF.^17^^,^^18^ Scheel et al^20^ reported that 12% of individuals enrolled in the Johns Hopkins ARVC registry ultimately underwent heart transplantation. These observations highlight the critical need for international collaboration to investigate HF-related outcomes in ACM. Such efforts should aim to elucidate disease-specific determinants of HF progression, establish evidence-based timing for advanced HF evaluation, and optimize strategies for and develop approaches to transplant waitlisting and qualification in ACM.

The contributions of the ChinaCORE ACM Registry and the Brazilian cohort underscore the essential role of regional studies in advancing our understanding of arrhythmogenic cardiomyopathy. These registries demonstrate that populations from different regions may exhibit unique patterns of disease expression, clinical progression, and risk factors. Such findings emphasize the importance of global collaboration and the inclusion of diverse populations in research efforts to improve diagnosis, risk stratification, and management of ACM worldwide.

## Funding Support and Author Disclosures

The authors have reported that they have no relationships relevant to the contents of this paper to disclose.
